# Supplementary material for: Saccharomyces boulardii (CNCM I-745) improves intestinal damage in sepsis by remodeling intestinal flora structure
Source: Front Cell Infect Microbiol. 2025 Jul 31;15:1602792. doi: 10.3389/fcimb.2025.1602792 (PMC12350329; doi:10.3389/fcimb.2025.1602792)
Supplement: Supplementary file 1 [file DataSheet1.zip › summary/summary/8_Advanced_analyse/2_PICRUSt2_result/METACYC/Group.BW4_vs_BW0/PICRUSt2_METACYC_Group.BW4_vs_BW0_diff.pdf]

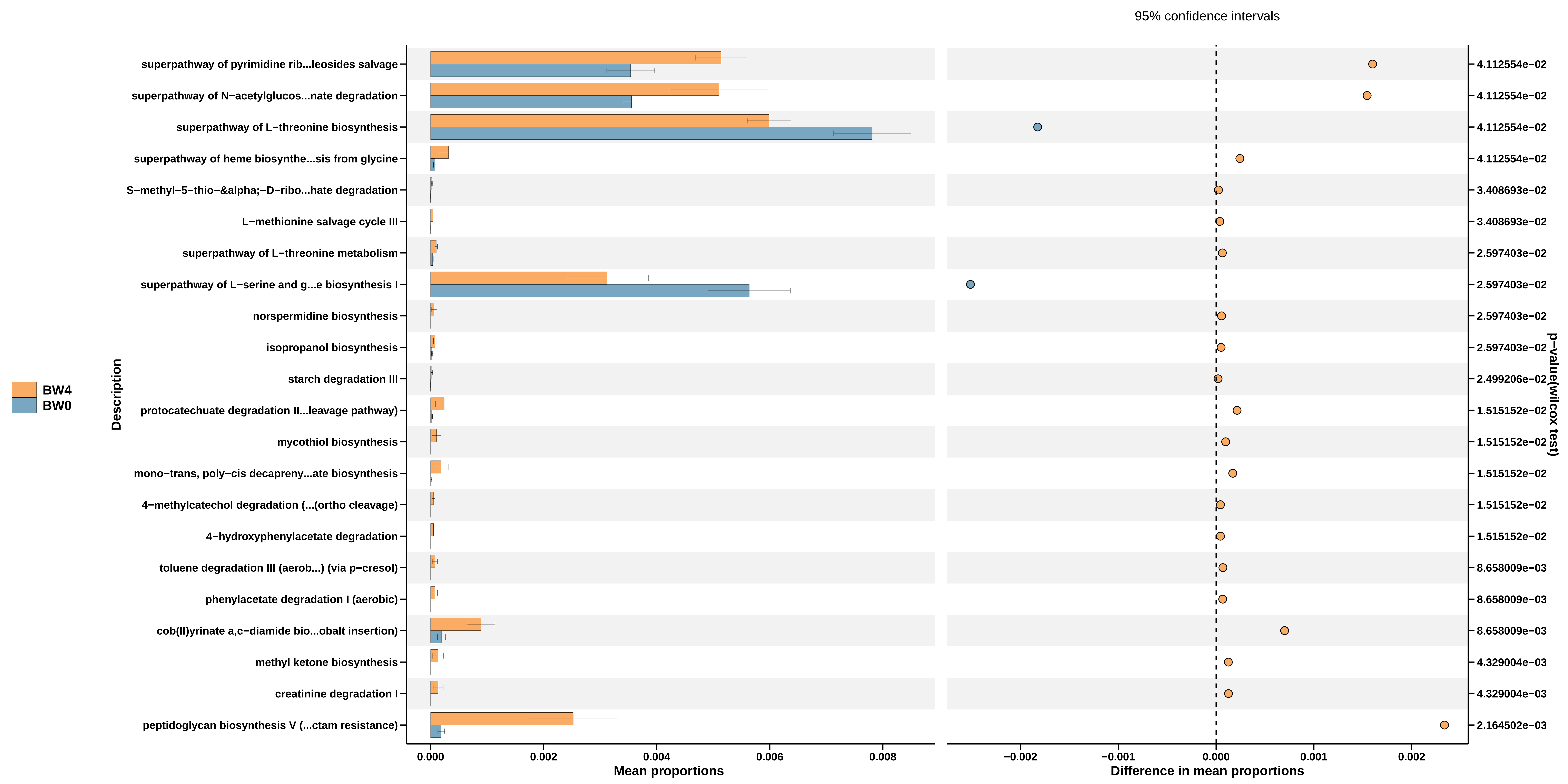
95% confidence intervals
BW4
BW0
Description
superpathway of pyrimidine rib...leosides salvage
superpathway of N−acetylglucos...nate degradation
superpathway of L−threonine biosynthesis
superpathway of heme biosynthe...sis from glycine
S−methyl−5−thio−α−D−ribo...hate degradation
L−methionine salvage cycle III
superpathway of L−threonine metabolism
superpathway of L−serine and g...e biosynthesis I
norspermidine biosynthesis
isopropanol biosynthesis
starch degradation III
protocatechuate degradation II...leavage pathway)
mycothiol biosynthesis
mono−trans, poly−cis decapreny...ate biosynthesis
4−methylcatechol degradation (...(ortho cleavage)
4−hydroxyphenylacetate degradation
toluene degradation III (aerob...) (via p−cresol)
phenylacetate degradation I (aerobic)
cob(II)yrinate a,c−diamide bio...obalt insertion)
methyl ketone biosynthesis
creatinine degradation I
peptidoglycan biosynthesis V (...ctam resistance)
0.000
0.002
0.004
0.006
0.008
Mean proportions
−0.002
−0.001
0.000
0.001
0.002
Difference in mean proportions
p−value(wilcox test)
4.112554e−02
4.112554e−02
4.112554e−02
4.112554e−02
3.408693e−02
3.408693e−02
2.597403e−02
2.597403e−02
2.597403e−02
2.597403e−02
2.499206e−02
1.515152e−02
1.515152e−02
1.515152e−02
1.515152e−02
1.515152e−02
8.658009e−03
8.658009e−03
8.658009e−03
4.329004e−03
4.329004e−03
2.164502e−03
